# The Role of Wnt/β-Catenin Pathway Mediators in Aortic Valve Stenosis

**DOI:** 10.3389/fcell.2020.00862

**Published:** 2020-09-10

**Authors:** Kashif Khan, Bin Yu, Chrystina Kiwan, Yousif Shalal, Sabin Filimon, Megan Cipro, Dominique Shum-Tim, Renzo Cecere, Adel Schwertani

**Affiliations:** Division of Cardiology and Cardiac Surgery, McGill University Health Centre, Montreal, QC, Canada

**Keywords:** human, calcification, immunohistochemistry, real-time qPCR, western blot, proteomics

## Abstract

Aortic valve stenosis (AVS) is a prevailing and life-threatening cardiovascular disease in adults over 75 years of age. However, the molecular mechanisms governing the pathogenesis of AVS are yet to be fully unraveled. With accumulating evidence that Wnt signaling plays a key role in the development of AVS, the involvement of Wnt molecules has become an integral study target in AVS pathogenesis. Thus, we hypothesized that the Wnt/β-catenin pathway mediators, SFRP2, DVL2, GSK3β and β-catenin are dysregulated in patients with AVS. Using immunohistochemistry, Real-Time qPCR and Western blotting, we investigated the presence of SFRP2, GSK-3β, DVL2, and β-catenin in normal and stenotic human aortic valves. Markedly higher mRNA and protein expression of GSK-3β, DVL2, β-catenin and SFRP2 were found in stenotic aortic valves. This was further corroborated by observation of their abundant immunostaining, which displayed strong immunoreactivity in diseased aortic valves. Proteomic analyses of selective GSK3b inhibition in calcifying human aortic valve interstitial cells (HAVICs) revealed enrichment of proteins involved organophosphate metabolism, while reducing the activation of pathogenic biomolecular processes. Lastly, use of the potent calcification inhibitor, Fetuin A, in calcifying HAVICs significantly reduced the expression of Wnt signaling genes Wnt3a, Wnt5a, Wnt5b, and Wnt11. The current findings of altered expression of canonical Wnt signaling in AVS suggest a possible role for regulatory Wnts in AVS. Hence, future studies focused on targeting these molecules are warranted to underline their role in the pathogenesis of the disease.

## Introduction

Aortic valve stenosis (AVS) is a prevailing cause of cardiac debility and death, affecting 2.8% of adults ≥75 years of age (Benjamin et al., 2011). It is associated with multiple risk factors such as hypertension, lack of exercise, hypercholesterolemia, diabetes and elevated plasma Lp(a) (Lindman et al., 2016). Aortic valvular narrowing, characterized by leaflet calcification and stiffening, eventually induces left ventricular obstruction and causes ischemic injury to the heart and brain (Greve et al., 2014; Spampinato et al., 2017). Therapies for AVS patients are limited to valve replacement surgery, with no treatment available to reverse the effects of the disease (James Everett et al., 2018). Therefore, examining the molecular signalling involved in the pathogenesis of AVS will aid in identifying more treatment modalities for this perilous disease.

The Wingless (Wnt) signaling pathway, implicated in a myriad of pathologies including cancer, neurodegenerative diseases, osteoporosis and several cardiovascular diseases (Nusse and Clevers, 2017; Zhan et al., 2017; Foulquier et al., 2018). Wnt ligands are a family of 19 secreted glycoproteins that signal through various pathways, depending on the binding of these ligands to specific Frizzled receptor isoforms on the plasma membrane (Albanese et al., 2018). The best understood is the canonical Wnt (Wnt/β-catenin) pathway, which promotes the nuclear translocation and stabilization of β-catenin, a transcriptional coactivator and intracellular signal transducer in the pathway (MacDonald et al., 2009). Canonical Wnt ligands bind to Frizzled cell surface receptors and lipoprotein-related peptide 5/6 (LRP5/6) co-receptors, activating the intracellular mediator, disheveled (DVL). This inhibits glycogen synthase kinase 3β (GSK-3β)-mediated phosphorylation of β-catenin by disruption of the APC/Axin/GSK3β complex. The accumulation of β-catenin in the nucleus ignites a gene transcription response by binding to the lymphoid enhancement factor/T-cell factor (LEF/TCF), activating genes involved in cell proliferation, differentiation and migration (Miller et al., 2013).

There is growing evidence for the association between Wnt signaling and the pathogenesis of AVS (Rajamannan, 2011, 2012; Albanese et al., 2018; Foulquier et al., 2018). Caira et al. (2006) were one of the first groups to show endochondral bone formation in the pathogenesis of AVS, which was associated with expression of canonical Wnt3a and coreceptors LRP5/6 in human calcified aortic valves. Secreted Wnt modulators Frizzled related protein-3, dickkops-1 and Wnt inhibitor factor-1 have been found to be elevated in the plasma of patients with AVS (Askevold et al., 2012). In addition, β-catenin has been shown to have pro-stenotic and pro-calcific effects in the pathogenesis of AVS (Miller et al., 2019). In a previous study from our laboratory, we suggested a critical role for the non-canonical Wnt ligands Wnt5a, Wnt5b, and Wnt11 in AVS. Aortic valve interstitial cells (VICs) treated with these Wnt ligands exhibited significant apoptosis and enhanced calcification (Albanese et al., 2016b). This likely to be mediated through frizzled and LRP5/6 co-receptor activation in calcified aortic valve leaflets, which play overlapping roles in Wnt/β-catenin signaling (Siddique et al., 2017). Interestingly, Wnt-signaling appears to play similar pathogenic and pro-calcific roles in multiple cell types during AVS development including HAVICs (Fang et al., 2018), endothelial cells (Masckauchán et al., 2005) and macrophages (Blumenthal, 2006). Yet, whether the level of some of the hallmark canonical Wnt/β-catenin mediators is altered remains unclear. In the present study, we utilize immunohistochemical (IHC) analyses, Real-Time qPCR and Western blotting to investigate the expression of GSK-3β, DVL, β-catenin, and SFRP2 in harvested human calcified aortic valves.

## Materials and Methods

### Tissue Collection

A total of 69 aortic valves were collected during cardiac valve replacement surgeries, along with a detailed clinical history (Table 1). Tissues were fixed in formalin and embedded in paraffin. Six normal aortic valves (mean age 53 ± 19; 3 males) from heart transplant donors, and 24 stenotic aortic valves were additionally collected during surgery for valve replacement, snap frozen in liquid nitrogen and stored at −80°C until processed for qPCR. The majority of patients with AVS had severe heart failure (NYHA class III–IV) with preserved ejection fraction. The study was approved by the ethics committee of the McGill University Health Centre, and informed consent was obtained from all participants involved in this study.

**TABLE 1:** Clinical data of patients with aortic valve disease used in immunohistochemistry.

**Clinical parameter**	**Control (*N* = 21) (Mean ± *SD*)**	**AVS (*N* = 48) (Mean ± *SD*)**
Age (years)	61.2 ± 11.7	79.8 ± 11.2*
Female (%)	38.1	37.5
HgA1c (%)	1.6 ± 2.7	6.2 ± 1.1*
Total cholesterol (mmol/L)	4.0 ± 1.2	3.6 ± 1.0
LDL (mmol/L)	2.2 ± 1.0	1.9 ± 0.8
HDL (mmol/L)	1.0 ± 0.3	1.1 ± 0.3
Triglycerides (mmol/L)	1.6 ± 0.7	1.2 ± 0.6^a^
Creatinine (μmol/L)	85.6 ± 30.1	100.7 ± 66.4
Albumin (mmol/L)	28.2 ± 7.5	30.7 ± 8.3
Circulating calcium (mmol/L)	2.0 ± 0.3	2.1 ± 0.2^b^
Aortic valve area (c*m*^2^)	2.3 ± 0.7	0.8 ± 0.3*
Pmax (mmHg)	13.1 ± 12.3	83.2 ± 28.2*
Pmean (mmHg)	6.8 ± 8.1	51.1 ± 18.7*
Jet velocity (cm/s)	169.5 ± 66.8	443.7 ± 80.0*
LVEF (%)	62.7 ± 6.5	56.4 ± 15.5

^a^P < 0.05 by sex, ^*b*^P < 0.05 by age, * P < 0.0001 compared to normal valves. AVS, aortic valve stenosis. * P < 0.05, ** P < 0.01. SD, standard deviation; HgA1c, hemoglobin A1c; LDL, low-density lipoprotein; HDL, high-density lipoprotein; Pmax, max pressure across the aortic valve; Pmean, mean pressure across the aortic valve; LVEF, left ventricular ejection fraction.

### mRNA Expression Analysis

Total RNAs were extracted from snap frozen aortic valve tissues using TRIzol (Invitrogen)/RNeasy Mini Kit (QIAGEN) combining protocol as previously described (Yu et al., 2017). Briefly, the first-strand cDNAs were synthesized using 1 μ*g* total RNAs with iScript^TM^ cDNA Synthesis Kit (Bio-Rad), the Real-Time qPCR were done using Advanced supergreen qPCR mastermix (Wisent Bioproducts) on StepOnePlus^TM^ Real-Time PCR System (Applied Biosystems^TM^). The data was analyzed using PrimePCR Analysis (Bio-RAD), and the normalized relative mRNA expression was calculated against GAPDH expression. Sequences of gene-specific primers are listed in Supplementary Table S1. Total RNAs were also extracted from cultured HAVICs for all *in vitro* mRNA expression experiments (*n* = 6).

### Immunohistochemistry

The paraffin-embedded tissue blocks were cut into 4 μ*m* sections using a microtome. The sections were incubated for 1 h in 10% normal goat serum/PBS solution, then incubated overnight with the primary antibodies in 0.1% BSA/PBS solution in humid chambers at 4°C. Primary antibodies used were rabbit anti-human GSK3β (GeneTex, at 1:200), DVL2 (GeneTex, at 1:200), β-catenin (Sigma-Aldrich, at 1:200) and SFRP2 (GeneTex, at 1:500). Secondary biotinylated goat anti-Rabbit IgG (Vector Labs, at 1:200) were applied followed by Vectastain ABC complex (Vector Laboratories, CA, United States) according to manufacturer protocol. Immunostaining was visualized by 1 × DAB/H_2_O_2_ solution, subsequently counterstained with hematoxylin, and mounted with Permount (Sigma–Aldrich). Co-localization studies were determined by immunostaining of proteins in regions of interest in consecutive 4 μ*m* sections. Immunostaining without primary antibody or with the primary antiserum preabsorbed with its respective antigen was carried out as negative control. Areas of lipids, calcification and endothelial cells were identified using Oil Red O, Alizarin Red, and von Willebrand Factor immunostaining, respectively (data not shown).

Aortic valves were semi-quantitatively scored in a blinded manner for overall immunostaining based on intensity and distribution throughout the valve (Supplementary Table S2). Aortic valves were also assessed for overall level of calcification, fibrosis, remodeling (Warren and Yong, 1997), and inflammation (Supplementary Table S3). The relationship between GSK-3β, DVL, β-catenin, and SFRP2 immunostaining and aortic valve histopathological feature was assessed by comparing the scoring of each tissue.

### Western Blotting

Calcified and non-calcified aortic valve tissues were lysed and re-suspended in RIPA buffer supplemented with cOmplete^TM^, Mini, EDTA-free Protease Inhibitor Cocktail (Sigma-Aldrich) for protein extraction. Samples were then centrifuged at 15,000 rpm at 4°C for 10 min and supernatants were collected, protein concentrations were measured by Protein DC protocol (Bio-Rad) and equal amount of total protein were loaded for 10% SDS-PAGE gel separation. The protein was transferred onto PVDF membrane at 300 mA for 1 h at RT, blocked for 1 h at room temperature with 5% milk (1 × TBST buffer), then incubated with primary antibodies against GSK3β (GeneTex, GTX111192 at 1:1000), DVL2 (GeneTex, GTX103878 at 1:1000) β-catenin (Sigma-Aldrich, HPA029159 at 1:1000) phosphorylated β-catenin (Abcam, ab11350 at 1:1000) and SFRP2 (GeneTex, GTX111892 at 1:1000) for 16 h at 4°C; then followed by incubation with corresponding secondary antibodies: bovine anti-mouse, or anti-rabbit IgG-HRP (Santa Cruz Biotechnology, sc-2370 and sc-2371, respectively, at 1:3000). For signal development, Lumi-Light Western Blotting Substrate (Sigma-Aldrich) was used according to manufacturer protocols.

### Isolation and Culture of HAVICs

Primary HAVICs were generated from healthy subjects as previously described (Al Kindi et al., 2014; Albanese et al., 2016a). Cultured HAVICs showed positive staining for alpha smooth muscle actin, indicating myofibroblast phenotype after 2 passages (data not shown). HAVICs at passages 3–5 were used for all experiments.

### Proteomic Profiling

Total proteins were extracted from HAVICs incubated in osteogenic media in the presence and absence of 10 μM CHIR99021 for 3 weeks. The media and drug were replaced every 2–3 days. The cells were lysed using RIPA lysis buffer containing protease inhibitor and centrifuged to remove cell debris. Protein quantification was performed using a DC assay. Prior to digestion, detergents were removed from samples using Pierce Detergent removal column (Thermo Fisher Scientific, Waltham, United States). Total proteins (50 μg) were diluted in in a solution containing 8 M urea, 10 mM HEPES-KOH (pH 7.5) and 5 mM DTT, heated for 2 min at 95°C, followed by an incubation of 30 min at room temperature. The samples were then alkylated, in the dark, for 30 min at room temperature by adding chloroacetamide to a final concentration of 7.5 mM (Sigma-Aldrich, Saint-Louis, United States). Urea was then decreased to 2 M by adding 3 volumes of 50 mM NH4HCO3. The proteins were then digested by adding 1 μg of Pierce MS-grade trypsin (Thermo Fisher Scientific, Waltham, United States) and incubated overnight at 30°C. Digestion was stopped by adding trifluoroacetic acid (TFA) (Sigma-Aldrich, St. Louis, United States) to a final concentration of 0.2%. The peptides were then purified with ZipTip 100-μl micropipet tips containing a C18 column (EMD Millipore, Burlington, United States), eluted 3 times with a solution containing 50% acetonitrile, 1% formic acid (FA) (Thermo Fisher Scientific, Waltham, United States). The eluted peptides were then concentrated by centrifugal evaporator at 65°C until complete drying and then resuspended in 25 μl of 1% FA. Peptides were assayed using a NanoDrop spectrophotometer (Thermo Fisher Scientific, Waltham, United States) and read at an absorbance of 205 nm. The peptides were then transferred to a glass vial (Thermo Fisher Scientific, Waltham, United States) and stored at −20°C until analysis by mass spectrometry.

### LC-MS/MS Analysis

Trypsin-digested peptides were separated by LC-MS/MS. Samples (250 ng of digested peptides) were injected into an HPLC (nanoElute, Bruker Daltonics) and loaded onto a trap column (Acclaim PepMap100 C18 column, 0.3 mm id × 5 mm, Dionex Corporation) with a constant flow of 4 μl/min and eluted onto an analytical C18 Column (1.9 μm beads size, 75 μm × 25 cm, PepSep). Peptides were eluted over a 2-hr gradient of acetonitrile (5–37%) in 0.1% FA at 500 nL/min while being injected into a TimsTOF Pro Mass Spectrometer equipped with a ZDV sprayer source (Bruker Daltonics). Data was acquired using data-dependent auto-MS/MS with a 100–1700 m/z mass range, with PASEF enabled a number of PASEF scans set at 10 (1.27 seconds duty cycle) and a dynamic exclusion of 0.4 min, m/z dependent isolation window and collision energy of 42.0 eV. The target intensity was set to 20,000, with an intensity threshold of 2,500.

### Protein Identification by MaxQuant Analysis

The raw files were analyzed with MaxQuant (version 1.6.10.43) (Cox and Mann, 2008; Tyanova et al., 2016) by using embedded tims-DDA specific parameters and the Uniprot human database (10/04/2018, 74,811 entries). The settings used for the MaxQuant analysis were: 2 miscleavages were allowed, fixed modification was carbamidomethylation on cysteine, enzymes were Trypsin (K/R not before P). Variable modifications included in the analysis were methionine oxidation, protein N-terminal acetylation and carbamylation (K, N-term). Label-free quantification with LFQ minimum ratio count of 2, identification values “PSM FDR”, “Protein FDR”, and “Site decoy fraction” of 0.05 were also used during analysis. Proteins positive for at least one of the “Reverse” and “Potential contaminant” categories were subsequently eliminated.

### Measurement of Calcium Mineralization

Alizarin Red S (ARS) staining was used to visualize the calcium deposition and calcification nodule formation as previously described (Reynolds et al., 2005). Briefly, HAVICs were incubated in OSM containing 2 mM phosphate and 1.8 mM calcium in 1% BSA for 2 days and fixed for 30 min using 4% buffered formaldehyde at room temperature. Cells were then washed 4 times with excessive ddH_2_O, incubated with Alizarin Red S stain solution (40 mM, pH4.2) for 20 min at room temperature, and again washed 4 times with excessive ddH_2_O. Calcification nodules were observed under microscopy, and phase contrast images were taken with inverted microscopy. After removing the ddH_2_O, calcium bound ARS were extracted by incubating with 10% Cetylpyridinium chloride (Sigma) solution at room temperature for 60 min. Extracts were transferred into new 1.5 ml centrifuge tube, spun for 5 min at 12,000 *g* and supernatant was aliquot in 96-well reading plate for OD 405 nm reading on Spectra Photometer (BioTek). Cells were further washed twice with ddH_2_O and scraped into 1.5 ml centrifuge tube in cell lysis buffer (20 mM Tris HCl pH7.4 with 2%SDS, 0.2 M glycine), and heat treated at 80°C for 60 min with occasional vortex. A Crystal Violet (CV) staining protocol was used on the same set of fixed cells for ARS normalization. *N* = 6 was used for all ARS experiments.

### Immunocytochemistry

Human aortic valve interstitial cells were seeded into chamber slides (BD Falcon) and Fetuin A starved for 48 h after incubated in serum free DMEM medium. On the day of experimentation, cells were fixed with 4% paraformaldehyde (Electron Microscopy Sciences) and permeated with 0.5% Triton X-100 (Electron Microscopy Sciences). HAVICs were stained with anti-human Fetuin A antibody (Thermo Fisher Scientific, PA5-51594 at 1:500), and Donkey anti-rabbit IgG Alexa Flour 488 (Invitrogen, 710369 at 1:1000). Nucblue reagent and ActinRed 555 reagent (Invitrogen) were used for nuclear and F-actin stain, respectively. Images were obtained using Zeiss LSM 780 confocal microscope.

### Statistical Analysis

A generalized linear model was used to assess differences between all clinical parameters adjusting for age and sex. Multivariant analysis was used to test the association between the semi-quantitative scoring of the immunostaining of GSK-3β, DVL, β-catenin and SFRP2 and histopathological plaque feature including calcification, fibrosis, remodeling and inflammation. Unpaired Student’s *t*-test was used to assess statistical significance between age, sex and echocardiographic parameters between AVS and healthy subjects. Unpaired Student’s *t*-test was used to assess differences in mRNA and protein expression measurements of GSK-3β, DVL2, β-catenin and SFRP2 between stenotic and non-stenotic aortic valves. For proteomic profiling, student *t*-test was used to determine the statistical significance of differentially expressed proteins in both groups. GO and KEGG pathway analyses were done using STRING (Szklarczyk et al., 2019). All other statistical analyses were performed using RStudio version 1.1.463. Significance level was set at *P* < 0.05. Data are presented as mean ± SEM unless otherwise indicated.

## Results

### qPCR Analysis of GSK-3β, DVL2, β-Catenin, SFRP2 in AVS

**FIGURE 1:**
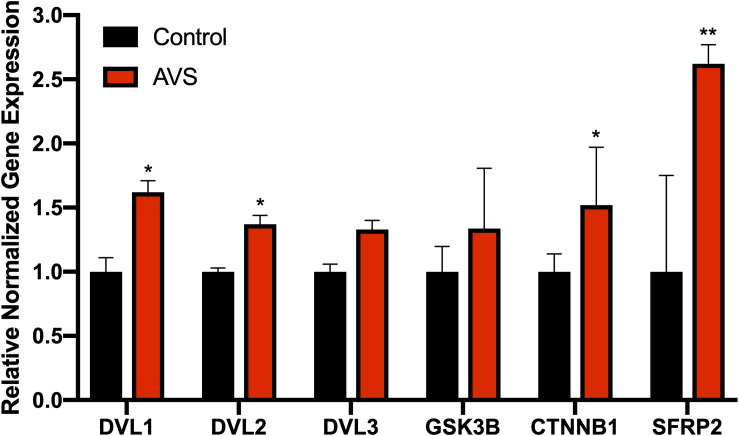
Stenotic aortic valves have greater mRNA expression of Wnt-signaling mediators compared to healthy valves. Increased mRNA expression was found for Wnt-signaling mediators DVL. AVS, aortic valve stenosis. The data are represented as mean ± SEM. Significance level was set at *P* < 0.05. *P < 0.05, **P < 0.01 compared to control groups; *n* = 31 for AVS samples and *n* = 5 for normal samples. Student’s *t*-test was used for statistical analysis.

The mRNA expression of DVL1 and DVL2 were significantly increased in stenotic valves compared to healthy valves, with no differences found for DVL3 (Figure 1). Additionally, stenotic valves had higher mRNA expression of β-catenin and SFRP2 (*P* < 0.05 and *P* < 0.01, respectively) compared to healthy valves. No significant differences were found for GSK3β mRNA expression.

### Immunohistochemical Analysis of DVL2

**FIGURE 2:**
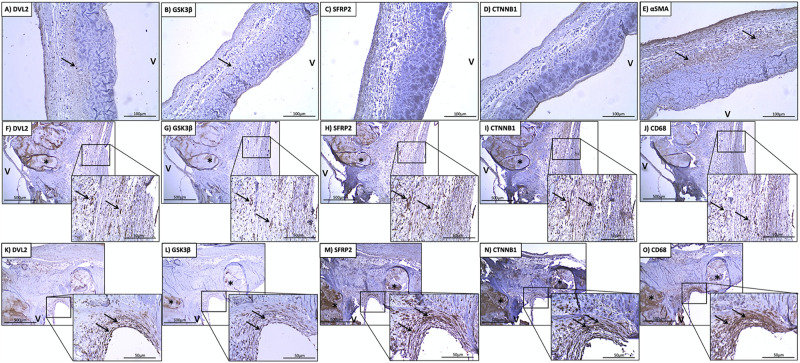
Stenotic aortic valves have greater immunostaining of Wnt/β-catenin mediators compared to healthy valves. Low **(A)** DVL2, **(B)** GSK-3β, **(C)** SFRP2, **(D)** β-catenin immunoreactivity observed in histologically normal leaflet (arrows indicate weak immunostaining in native cells). Prevalent immunostaining of **(E)** αSMA in histologically normal leaflets. Co-localization of **(F)** DVL2, **(G)** GSK-3β, **(H)** SFRP2 **(I)**, β-catenin immunoreactivity with macrophages around areas of calcification (arrows), indicated by **(J)** CD68 staining. Co-localization of **(K)** DVL2, **(L)** GSK-3β, **(M)** SFRP2 **(N)**, β-catenin immunoreactivity in valve interstitial cells in the thickened fibrosa (arrows), indicated by **(O)** αSMA staining. *Calcified foci; v, ventricular side of the leaflet. Arrows point to regions of interest.

There was little immunostaining for DVL2 in normal aortic valves (Figure 2A). More specifically, there was little immunostaining present in VICs on both the aorta and ventricular sides of the leaflet, with some staining found in the endothelium. DVL2 immunostaining was found in infiltrating macrophages along the endothelium and around areas of calcification (Figures 2F,J). There was also abundant DVL2 immunoreactivity found in the myointimal cells surrounding calcified regions (Figures 2K,O). Semi-quantitative analysis showed significant correlations between DVL2 and calcification (*r* = 0.5739, *P* < 0.0001), remodelling (*r* = 0.3889, *P* < 0.01), and inflammation (*r* = 0.3927, *P* < 0.01). No significant correlations were found for DVL2 and fibrosis. Histologically normal leaflets appeared to have significant αSMA immunostaining (Figure 2E). Negative control sections for DVL2 did not show any immunoreactivity.

### Immunohistochemical Analysis of GSK-3β

There was little-to-no immunostaining for GSK-3β found in normal aortic valves (Figure 2B). Significant immunostaining of GSK-3β was found in both infiltrating macrophages (Figures 2G,J) myointimal cells (Figures 2L,O) and around areas of fibrosis. Seldom GSK-3β immunostaining was found in the calcified foci (Figure 2L). This was also colocalized with immunostaining for αSMA (Figure 2O). Semi-quantitative analysis revealed significant correlations between GSK-3β and calcification (*r* = 0.4983, *P* = 0.001), fibrosis (*r* = 0.3113, *P* < 0.05), and remodelling (*r* = 0.4098, *P* < 0.01). No significant correlations were found for GSK-3β and inflammation. Negative control sections for GSK-3β did not show any immunoreactivity.

### Immunohistochemical Analysis of SFRP2

There was little immunostaining for SFRP2 found in normal aortic valves (Figure 2C). SFRP2 immunostaining was found in infiltrating macrophages in the fibrosa (Figures 2H,J). Very strong SFRP2 immunostaining was found in myointimal cells in areas of fibrosis and calcification (Figures 2M,O). Semi-quantitative analysis revealed significant correlations between SFRP2 and calcification (*r* = 0.5717, *P* < 0.0001), fibrosis (*r* = 0.3452, *P* < 0.05), and remodelling (*r* = 0.5174, *P* < 0.001). No significant correlations were found for SFRP2 and inflammation. Negative control sections for SFRP2 did not show any immunoreactivity.

### Immunohistochemical Analysis of β-Catenin

There was little immunostaining for β-catenin found in normal aortic valves (Figure 2D). β-catenin immunostaining was found in infiltrating macrophages along the endothelium (Figures 2I,J). Very strong β-catenin immunostaining was found in myointimal cells in areas of fibrosis (Figures 2N,O). Semi-quantitative analysis revealed significant correlations between β-catenin and calcification (*r* = 0.7689, *P* < 0.01). No significant correlations were found between β-catenin and fibrosis, remodelling, and inflammation. Negative control sections for β-catenin did not show any immunoreactivity.

### Western Blot of GSK-3β, DVL2, β-Catenin, and SFRP2

**FIGURE 3:**
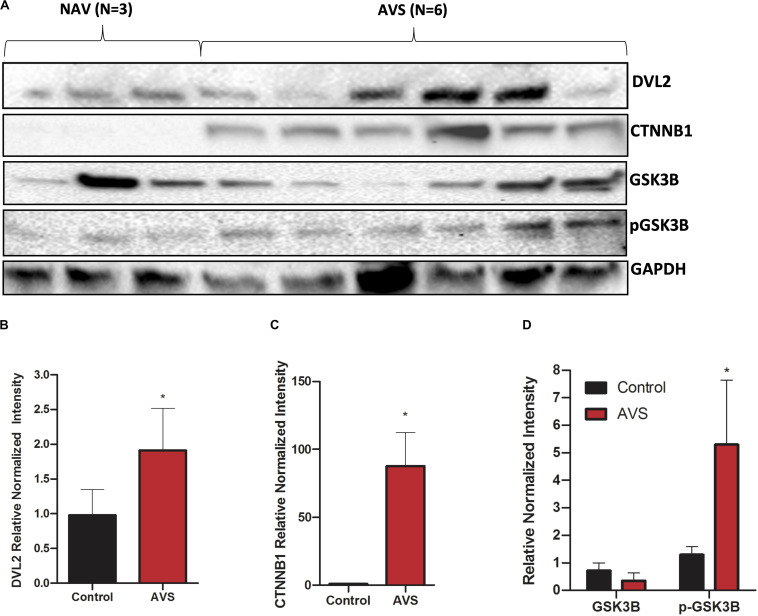
Stenotic aortic valves have greater protein expression of Wnt-signaling mediators compared to healthy valves. **(A)** Representative Western blot of DVL2, GSK-3β, p-GSK-3β, and of β-catenin protein expression in AVS patients compared to healthy patients. Densitometry analysis of DVL2 **(B)**, GSK-3β, p-GSK-3β **(C)**, and of β-catenin **(D)** protein expression. The data are represented as mean ± SEM. Significance level was set at *P* < 0.05. *P < 0.05 compared to control groups; *n* = 6 for AVS samples and *n* = 3 for normal samples. Students *t*-test was used for statistical analysis. AVS, aortic valve stenosis.

Western blotting showed stronger expression of DVL2 in stenotic valves compared to healthy tissues (Figures 3A,B, *P* < 0.05). β-catenin protein expression was only found in stenotic valves and was virtually undetectable in healthy valves (Figure 3C). No significant differences were found for GSK3β between healthy and stenotic valves (Figure 3D, *P* < 0.05). However, there was significantly higher expression of phosphorylated-GSK3β protein in stenotic valves compared to healthy valves, which could well explain the lack of difference in GSK3b mRNA expression. We could not detect any bands for SFRP2 in either AVS samples or healthy controls (data not shown).

### Proteomic Profiling of CHIR99021-Treated HAVICs

**FIGURE 4:**
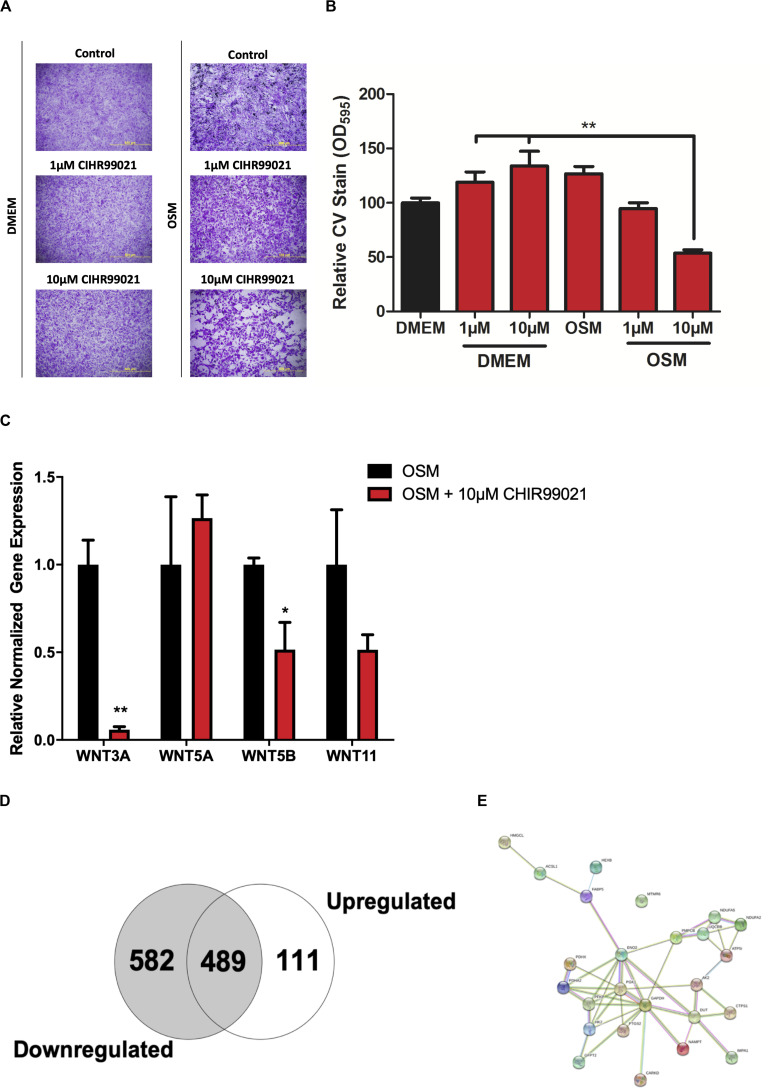
Proteomic profiling of HAVICs treated with CHIR99021. **(A)** Representative images of CV staining in HAVICs treated with CHIR99021 in DMEM or OSM. **(B)** Quantification of CV staining. The data are represented as mean ± SEM. Significance level was set at *P* < 0.05. *P < 0.05, **P < 0.01 compared to the specified group; *n* = 6 HAVIC lines were used for each group. One-way ANOVA followed by Tukey’s *post hoc* multiple comparisons test was used for statistical analysis. **(C)** mRNA expression of Wnt ligands after treatment with CHIR99021. The data are represented as mean ± SEM. Significance level was set at *P* < 0.05. *P < 0.05, **P < 0.01 compared to OSM groups; *n* = 3 HAVIC lines were used for each group. Students *t*-test was used for statistical analysis. **(D)** Venn diagram representing the number of differentially expressed proteins in HAVICs treated with CHIR99021 for 3 weeks in OSM compared to cells incubated in OSM alone. Fischer’s exact *t*-test was used for statistical analysis. **(E)** Network graphic of the highly enriched gene ontology biological process *organophosphate metabolic processes*. HAVICs, human aortic valve interstitial cells; CV, crystal violet; OSM, osteogenic media.

To investigate the mechanisms by which Wnt-signaling pathways promote AVS, we incubated HAVICs in osteogenic medium in the presence and absence of the GSK3β inhibitor CHIR99021, and investigated changes in the cell proteome. First, we have previously shown that treatment with CHIR99021 in HAVICs almost completely attenuates calcification (Albanese et al., 2016b). Treatment with CHIR99021 in DMEM did not significant change cell viability (Figures 4A,B). However, treatment with 10 μM CHIR99021 showed significant reductions in number of viable cells in OSM for 3 weeks (*P* < 0.01). This was accompanied by a reduction in mRNA expression of the canonical Wnt3a and non-canonical Wnt 5b ligands (Figure 4C, *P* < 0.01 and *P* < 0.05, respectively). Fischer’s exact t-test revealed a total of 693 differentially expressed proteins (DEPs), 111 of which were upregulated and 582 were downregulated (Figure 4D) in HAVICs treated with CHIR99021 in OSM compared to cells incubated in OSM alone. Gene Ontology (GO) analyses confirmed the downregulation of several Wnt-signaling pathways (Table 2) and the enrichment of several processes involved in metabolism (Table 3), including the *organophosphate metabolic processes* (Figure 4E). In addition, KEGG pathway analysis revealed the downregulation of several important biological pathways known to play a role in calcification and osteogenesis including the PI3K-AKT, MAPK, apoptosis, RAS, and JAK-STAT signaling pathways (Table 4).

**TABLE 2:** List of Wnt-signaling pathways downregulated in CHIR99021-treated HAVICs.

**GO term ID**	**Biological process**	**Observed protein count**	**FDR**	**Proteins**
GO:0030111	Regulation of Wnt signaling pathway	22	0.0016	APP, ATP6V0C, BICC1, CAV1, CCND1, COL1A1, DAB2, DDX3X, EGFR, EMD, GPC3, IGFBP2, ILK, ITGA3, JUP, LRP1, MACF1, PPAP2B, SEMA5A, SFRP4, SLC9A3R1, VCP
GO:0030177	Positive regulation of Wnt signaling pathway	13	0.0024	ATP6V0C, CAV1, COL1A1, DAB2, DDX3X, EGFR, GPC3, ILK, JUP, MACF1, SEMA5A, SFRP4, VCP
GO:0060828	Regulation of canonical Wnt signaling pathway	16	0.0111	APP, BICC1, CAV1, COL1A1, DAB2, DDX3X, EGFR, EMD, GPC3, IGFBP2, ILK, JUP, SEMA5A, SFRP4, SLC9A3R1, VCP
GO:0090263	Positive regulation of canonical Wnt signaling pathway	9	0.0212	CAV1, COL1A1, DDX3X, EGFR, ILK, JUP, SEMA5A, SFRP4, VCP

*HAVIC, human aortic valve interstitial cells. FDR, false discovery rate.*

**TABLE 3:** Top 10 enriched biological processes in CHIR99021-treated HAVICs.

**GO term ID**	**Biological process**	**Observed protein count**	**FDR**	**Proteins**
GO:0044281	Small molecule metabolic process	35	4.80E-08	ACSL1, AK2, AKR1B1, ATP5I, CARKD, CCBL2, CTPS1, DARS, DECR2, DHRS7B, DUT, ENO2, ETFB, FABP5, GAPDH, GFPT2, HEXB, HK2, HMGCL, IMPA1, MAN2B1, NAMPT, NDUFA2, NDUFA5, PDHA2, PDHX, PFKP, PGK1, PHGDH, PLOD2, PMPCB, PTGS2, SHMT2, TARS, UQCRB
GO:0006091	Generation of precursor metabolites and energy	17	1.06E-07	AKR1B1, ATP5I, ENO2, ETFB, GAPDH, GFPT2, HK2, HMGCL, NDUFA2, NDUFA5, NDUFAF2, PDHA2, PFKP, PGK1, PHGDH, PMPCB, UQCRB
GO:0009117	Nucleotide metabolic process	20	1.06E-07	ACSL1, AK2, ATP5I, CARKD, CTPS1, DUT, ENO2, GAPDH, HK2, HMGCL, NAMPT, NDUFA2, NDUFA5, PDHA2, PDHX, PFKP, PGK1, PMPCB, PTGS2, UQCRB
GO:0009165	Nucleotide biosynthetic process	15	1.06E-07	ACSL1, AK2, ATP5I, CARKD, CTPS1, DUT, ENO2, GAPDH, HK2, NAMPT, PDHA2, PDHX, PFKP, PGK1, PTGS2
GO:0019637	Organophosphate metabolic process	25	1.06E-07	ACSL1, AK2, ATP5I, CARKD, CTPS1, DUT, ENO2, FABP5, GAPDH, GFPT2, HEXB, HK2, HMGCL, IMPA1, MTMR6, NAMPT, NDUFA2, NDUFA5, PDHA2, PDHX, PFKP, PGK1, PMPCB, PTGS2, UQCRB
GO:0055086	Nucleobase-containing small molecule metabolic process	21	1.06E-07	ACSL1, AK2, ATP5I, CARKD, CTPS1, DUT, ENO2, GAPDH, GFPT2, HK2, HMGCL, NAMPT, NDUFA2, NDUFA5, PDHA2, PDHX, PFKP, PGK1, PMPCB, PTGS2, UQCRB
GO:0090407	Organophosphate biosynthetic process	19	1.76E-07	ACSL1, AK2, ATP5I, CARKD, CTPS1, DUT, ENO2, FABP5, GAPDH, HEXB, HK2, IMPA1, MTMR6, NAMPT, PDHA2, PDHX, PFKP, PGK1, PTGS2
GO:0009435	NAD biosynthetic process	8	2.87E-07	CARKD, ENO2, GAPDH, HK2, NAMPT, PFKP, PGK1, PTGS2
GO:1901566	Organonitrogen compound biosynthetic process	28	3.45E-07	ABCB6, ACSL1, AK2, ATP5I, CARKD, CHCHD1, CTPS1, DARS, DUT, EEF1A2, EEFSEC, EIF4EBP1, ENO2, FABP5, GAPDH, GFPT2, HK2, MRPL12, NAMPT, PDHA2, PDHX, PFKP, PGK1, PHGDH, PLOD2, PTGS2, SHMT2, TARS
GO:0007005	Mitochondrion organization	16	5.29E-07	AFG3L2, APOOL, ATP5I, HK2, HMGCL, LONP1, MIPEP, NDUFA2, NDUFA5, NDUFAF2, PMPCB, SAMM50, TIMM13, TIMM22, TOMM70A, UQCRB

*HAVIC, human aortic valve interstitial cells. FDR, false discovery rate.*

**TABLE 4:** List of downregulated KEGG pathways in CHIR99021-treated HAVICs known to play a role in the pathogenesis of AVS.

**KEGG term**	**KEGG pathway**	**Observed protein count**	**FDR**	**Proteins**
hsa04810	Regulation of actin cytoskeleton	31	1.36E-10	ACTN4, ARHGEF1, ARHGEF7, CFL2, CYFIP1, DIAPH1, EGFR, EZR, F2R, ITGA11, ITGA2, ITGA3, ITGA7, ITGA8, ITGAV, ITGB1, ITGB5, MAPK1, MYH10, MYH14, MYH9, MYL9, MYLK, PDGFRA, PDGFRB, PPP1R12A, PPP1R12C, RRAS, RRAS2, VCL, WASL
hsa04151	PI3K-Akt signaling pathway	33	8.06E-07	AKT1, CCND1, CDK4, COL1A1, COL1A2, COL4A1, COL6A1, COL6A2, COL6A3, EGFR, EPHA2, F2R, GNB4, HSP90AA1, HSP90AB1, HSP90B1, ITGA11, ITGA2, ITGA3, ITGA7, ITGA8, ITGAV, ITGB1, ITGB5, LAMC1, MAPK1, NTRK2, PDGFRA, PDGFRB, PRKCA, RHEB, RPS6, YWHAH
hsa05418	Fluid shear stress and atherosclerosis	14	0.0013	AKT1, ASS1, CAV1, GPC1, GSTM2, HSP90AA1, HSP90AB1, HSP90B1, ITGAV, NQO1, PLAT, SQSTM1, SUMO2, TXN
hsa03013	RNA transport	14	0.0054	CYFIP1, EIF2B4, EIF2S2, EIF3A, EIF3B, EIF3D, EIF3G, EIF3H, EIF3I, EIF5, EIF5B, RANGAP1, SUMO2, TPR
hsa04010	MAPK signaling pathway	18	0.0244	AKT1, CACNA2D1, EGFR, EPHA2, FLNA, FLNB, FLNC, HSPA2, HSPB1, MAPK1, MAPKAPK3, NTRK2, PDGFRA, PDGFRB, PRKCA, RRAS, RRAS2, TRADD
hsa04270	Vascular smooth muscle contraction	10	0.0247	ARHGEF1, CALD1, GNAS, MAPK1, MYL9, MYLK, PPP1R12A, PPP1R12C, PRKCA, PRKG1
hsa04210	Apoptosis	10	0.0427	AKT1, CAPN1, CAPN2, CTSD, CTSK, MAPK1, PARP4, SPTAN1, TRADD, TUBA4A
hsa04015	Rap1 signaling pathway	13	0.0441	AKT1, EGFR, EPHA2, F2R, GNAI2, GNAS, ITGB1, MAPK1, PDGFRA, PDGFRB, PRKCA, RRAS, TLN1
hsa04014	Ras signaling pathway	14	0.0454	AKT1, EGFR, EPHA2, EXOC2, GNB4, MAPK1, NTRK2, PDGFRA, PDGFRB, PRKCA, PTPN11, RRAS, RRAS2, TBK1
hsa04630	Jak-STAT signaling pathway	11	0.0454	AKT1, CCND1, EGFR, FHL1, IL13RA2, IL6ST, PDGFRA, PDGFRB, PTPN11, STAT2, STAT6

*AVS, aortic valve stenosis. FDR, false discovery rate.*

### Fetuin A Attenuates Calcification in HAVICs via Inactivation of Wnt Signaling Genes

**FIGURE 5:**
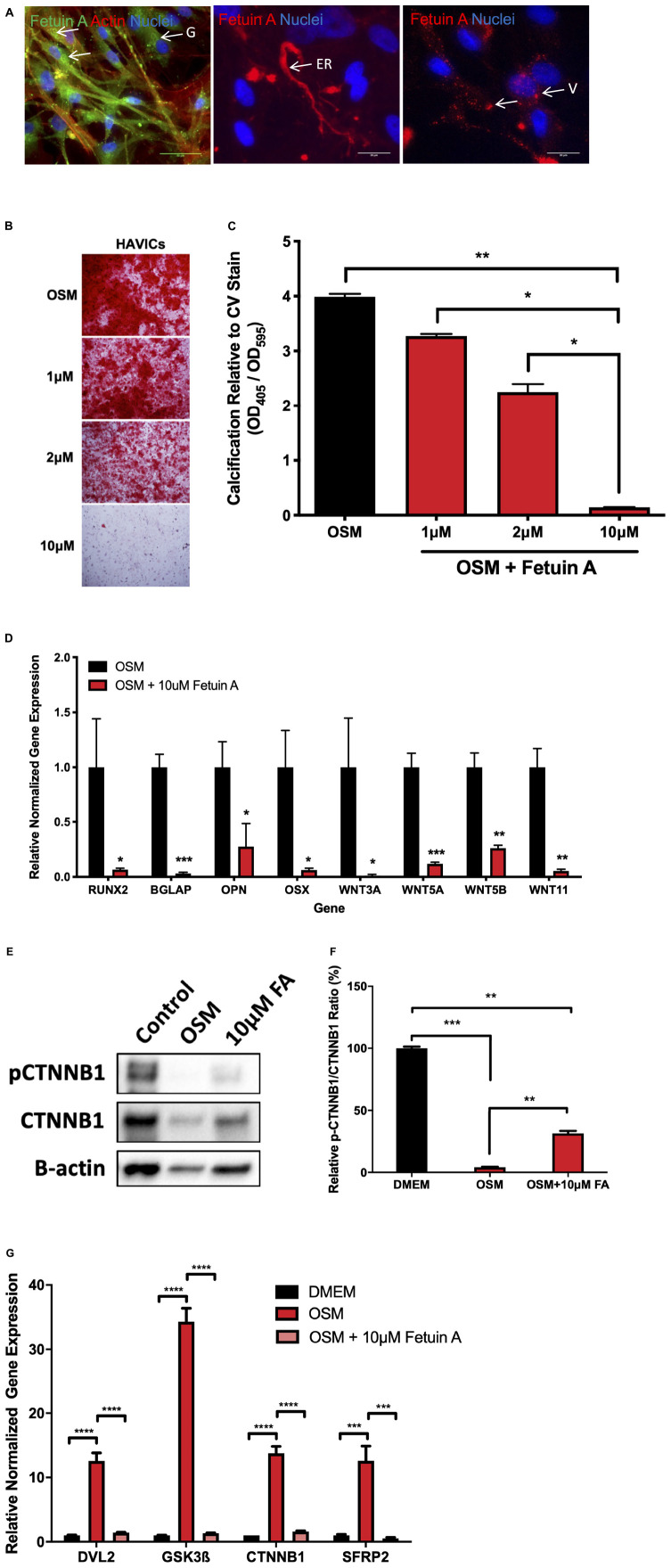
Fetuin A attenuates calcification in HAVICs and is associated with inactivation of Wnt signaling genes. **(A)** Representative images of endogenous Fetuin A in HAVICs localized to the golgi apparatus (G), endoplasmic reticulum (ER), and vesicles (V). Arrows point to regions of interest. Scale bar: 20 μm. **(B)** Representative Alizarin Red S staining images of HAVICs treated with different concentrations of Fetuin A. **(C)** Quantification of calcium deposition using acid extraction following Alizarin Red S stain of HAVIC relative to Crystal Violet stain. The data are represented as mean ± SEM. Significance level was set at *P* < 0.05. *P < 0.05, **P < 0.01 compared to the specified group; *n* = 6 HAVIC lines were used for each group. One-way ANOVA followed by Tukey’s *post hoc* multiple comparisons test was used for statistical analysis. **(D)** mRNA expression of osteogenic and Wnt signaling genes after treatment with Fetuin A. The data are represented as mean ± SEM. Significance level was set at *P* < 0.05. *P < 0.05, **P < 0.01, ***P < 0.001 compared to control groups; *n* = 6 HAVIC lines were used for each group. Students *t*-test was used for statistical analysis. **(E)** Representative Western blot of phosphorylated β-catenin and β-catenin protein expression in HAVICs treated with 10 μM Fetuin A in OSM. **(F)** Densitometry analysis of phosphorylated β-catenin and β-catenin protein expression in HAVICs treated with 10 μM Fetuin A in OSM. The data are represented as mean ± SEM. Significance level was set at *P* < 0.05. **P < 0.01, ***P < 0.001; *n* = 2 for each group. Students *t*-test was used for statistical analysis. **(G)** mRNA expression of intracellular Wnt signaling genes after treatment with Fetuin A. The data are represented as mean ± SEM. Significance level was set at *P* < 0.05. ***P < 0.001, ****P < 0.0001 compared to control groups; *n* = 3 HAVIC lines were used for each group. One-way ANOVA followed by Tukey’s *post hoc* multiple comparisons test was used for statistical analysis. AVS, aortic valve stenosis; HAVICs, human aortic valve interstitial cells; OSM, osteogenic media; FA, Fetuin A.

In order to further elucidate the role of Wnt signaling in AVS, we utilized Fetuin A, a potent calcification inhibitor and investigated changes in gene expression. Immunofluorescence staining of endogenous Fetuin A showed localization in the golgi apparatus, endoplasmic reticulum and cellular vesicles (Figure 5A). Utilizing an Alizarin Red S staining assay, treating HAVICs with Fetuin A significantly attenuated calcification onset in a concentration-dependent manner (Figures 5B,C, *P* < 0.0001). mRNA expression analyses confirmed significant downregulation of osteogenic genes runt-related transcription factor 2 (Runx2) (*P* < 0.05), bone Gamma-Carboxyglutamate Protein (BGLAP) (*P* < 0.0001), osteopontin (OPN) (*P* < 0.05) and osterix (OSX) (*P* < 0.05) after treatment with 10 μM Fetuin A (Figure 5D). This was accompanied by a similar reduction in the Wnt genes: Wnt3a (*P* < 0.05), Wnt5a (*P* < 0.0001), Wnt5b (*P* < 0.001), and Wnt11 (*P* < 0.001). HAVICs incubated in OSM for 48 h had reduced phosphorylated β-catenin/β-catenin protein expression ratio (Figures 5E,F, *P* < 0.001) and Fetuin A treatment was able to attenuate this response (*P* < 0.01). Additionally, Fetuin A reduced the mRNA expression of intracellular Wnt mediators DVL2, GSK3β, CTNNB1, and SFRP2 after incubation in OSM (Figure 5G, *P* < 0.001).

## Discussion

Emerging evidence testifies to the involvement of the canonical Wnt signaling pathway in the pathogenesis of AVS (Alfieri et al., 2010; Askevold et al., 2012; Mathieu et al., 2014; Foulquier et al., 2018). Our lab has previously demonstrated that several non-canonical Wnt ligands are present in AVS patients and promote calcification and apoptosis in HAVICs (Albanese et al., 2017). Additionally, we showed that the mRNA and protein expression of frizzled receptors and their co-receptors are upregulated in AVS patients (Siddique et al., 2017). Accordingly, interest in the modulators by which Wnt signaling is regulated is growingly ignited. In this study, we show the expression and localization of four Wnt-signaling molecule DVL2, GSK3β, β-catenin, and SFRP2, in AVS. All four proteins were abundantly expressed in stenotic aortic valves. In addition, qPCR analysis showed upregulation of DVL1, DVL2, β-catenin, and SFRP2 mRNA in stenotic aortic valves compared to healthy tissues. Together, our results clearly demonstrate upregulation of four key Wnt-signaling modulators in AVS patients, suggesting a possible role for the system in AVS.

The regulatory role of GSK-3β in Wnt signaling is controversial and largely depends on the type of cell and environment. Although there is a consensus in the literature suggesting that phosphorylation of GSK-3β at Ser9 (pSer9-GSK-3β) is inactivating and promotes formation of the destruction complex, several studies suggest the opposite, where pSer9-GSK-3β is a constitutively active form that promotes osteogenic differentiation and matrix mineralization (Huh et al., 2013). In fact, both theories may be true: only a small portion of pSer9-GSK-3β is bound to the destruction complex, while the rest play other regulatory roles in the cytoplasm (Beurel et al., 2015). Several signaling pathways such as PI3K-AKT, insulin, and other G-protein coupled receptor-mediated pathways converge on to GSK-3β, indicating its function may be more complicated than simply acting as a Wnt signaling activator. In this study, we found no changes in mRNA or protein expression of GSK-3β in healthy and stenotic aortic valves, however, the expression of pSer9-GSK-3β was significantly increased in AVS and expressed in areas of calcification, fibrosis and lipid core. These findings are corroborated by our previous results, where human aortic valve interstitial cells treated with CHIR99021, a potent GSK-3β inhibitor, had significantly reduced calcium deposition under osteogenic conditions (Albanese et al., 2016b). Together, our study supports the idea that pSer9-GSK-3β plays a pathogenic role in AVS that is independent of Wnt signaling.

Changes in calcium and phosphate metabolism have been largely associated with calcification of the aortic valve (Yang et al., 2015). In fact, previous studies have shown that patients with AVS have elevated serum calcium, phosphate and parathyroid hormone levels with reduced 25-hydroxyvitamin (Mills et al., 2004; Linhartová et al., 2008), suggesting impairments in mineral metabolism. However, the mechanisms by which this occurs remains to be elucidated. In this study, we mineralized HAVICs with the addition of phosphate to the cell growth medium, which we have previously demonstrated to induce extensive mineralization (Albanese et al., 2017). In the same study, GSK-3β inhibition significantly reduced mineralization after 3 weeks. Our proteomics analysis suggests that the majority of enriched biological process that were activated are largely associated with changes in metabolism, namely *organophosphate metabolic processes.* One of the proteins that were largely enriched was nicotinamide phosphoribosyltransferase (Nampt), which plays a critical role in the regulation of plasma inorganic phosphate levels in part through maintaining nicotinamide adenine dinucleotide (NAD) homeostasis (Miyagawa et al., 2018). Interestingly, inhibiting Nampt in wild-type mice reduced inorganic phosphate excretion. Another study showed the Nampt inhibition dampened glycolysis, which is an important mediator of phosphate metabolism (Tan et al., 2015).

Fetuin A, formally known as α2-Heremans–Schmidt glycoprotein, is described as a “major systemic inhibitor of ectopic calcification” (Jahnen-Dechent et al., 2008). Mice that are deficient in Fetuin A display microcalcification features in their small vessels along with generalized connective tissue calcification (Schäfer et al., 2003), while treating vascular smooth muscle cells with Fetuin A has been shown to robustly attenuate calcification (Reynolds et al., 2005). Here, we are the first to show that Fetuin A reduces calcification in HAVICs at concentrations similar to that which is found in the serum. Interestingly, the reduced calcification seen in the HAVICs was accompanied by a nearly complete reduction in mRNA expression of Wnt3a, Wnt5a, Wnt5b, and Wnt11. In a previous study, we show that the non-canonical proteins Wnt5a, Wnt5b, and Wnt11 are differentially expressed in patients with AVS, and that treating HAVICs with these ligands drives calcification (Albanese et al., 2017). Runx2 has been shown to be a master transcriptional regulator of osteogenic differentiation and is regulated directly by Wnt signaling (Gaur et al., 2005; Albanese et al., 2018). Here, we also show that the reduced expression of Wnt-signaling genes is accompanied by a reduction in RUNX2, as well as other osteogenic genes such as OPN, OSX, and BGLAP. These results are further validated by the ability of Fetuin A to increase the phosphorylated β-catenin to β-catenin protein expression ratio after during incubation in OSM, indicating downregulation of the active form of β-catenin (Albanese et al., 2018; Houschyar et al., 2019). Furthermore, Fetuin A completely attenuated the increased mRNA expression seen for DVL2, GSK3β, β-catenin, and SFRP2 in calcifying HAVICs. Together, these results confirm a procalcifying role for Wnt signaling in HAVICs and that Fetuin A may be a potential therapeutic to prevent calcification.

It is likely the pathogenesis of AVS is orchestrated by several biological pathways that work together to initiate changes in gene transcription and cell function. Our GO analysis coincides with these studies, suggesting that inhibiting GSK-3β initiates a cascade of events, leading to the downregulated profibrocalcifying mechanisms such as the Janus kinase-signal transducer and activator of transcription (Parra-Izquierdo et al., 2018), Ras-signaling (Kumar et al., 2014), shear stress (Wang et al., 2013), and apoptosis (Leopold, 2012; Galeone et al., 2013) pathways. This data coincides with our previous studies, showing that lipoprotein(a)-induced valvular calcification occurs through the activation of several biological pathways (Yu et al., 2017, 2018). Of particular interest, we found that Calreticulin was downregulated in CHIR99021-treated HAVICs. Calreticulin is a chaperone protein that binds to misfolded protein to prevent their export from the endoplasmic reticulum (Eggleton et al., 2016). Additionally, Calreticulin has been associated with AVS, along with other cardiovascular diseases (De La Cuesta et al., 2009; Martín-Rojas et al., 2012).

SFRPs may activate or inactivate Wnt-signaling and therefore, their role in the pathogenesis of AVS is currently unknown and remains controversial in other cardiac pathologies as well (Bovolenta et al., 2008; Foulquier et al., 2018). Of these molecules, SFRP2 is the most widely studied in the context of cardiac development and disease (Foulquier et al., 2018; Wu et al., 2020). For example, in animal models of myocardial infarction, SFRP2 gene deletion attenuated fibrosis and improved cardiac function, while treatment with SFRP2 antibody reduced apoptosis and fibrosis (Kobayashi et al., 2009; Mastri et al., 2014). Other reports have shown that SFRP2 is a potent inhibitor of chondrogenesis (Morello et al., 2008; Woldt et al., 2012), warranting further investigation of this important Wnt modulator in the context of AVS. In the current study, we show upregulation of SFRP2 mRNA, and localization of SFRP2 protein in foam cells and calcification cores. This may be explained through the ability of SFRPs to form complexes with each other to impede their ability to scavenge Wnt ligands, or by directly binding to Frizzled receptors to block Wnt binding (Bovolenta et al., 2008). In cardiac fibroblasts, SFRP2 activated canonical Wnt signaling to activate matrix metalloproteinases and accelerate extracellular matrix remodelling (Lin et al., 2016). Additionally, injections of a SFRP2 antibody in a failing hamster heart attenuated Wnt-signaling while also reducing fibrosis and improving cardiac function (Mastri et al., 2014). Still, it is not clear if SFRP2 is contributing to AVS progression, or if SFRP2 is released by resident cells to attenuate Wnt-signaling and valve remodeling. Further research in this area may help clarify these findings.

In summary, the present study demonstrates the increased expression of GSK-3β, DVL2, SFRP2, and β-catenin stenotic aortic valves. We also identified some of the pathways involved in the maladaptive changes in HAVICs associated with GSK-3β activation. These findings may propel research aimed at targeting molecules involved in the canonical Wnt pathway in order to eventually develop potentially propitious pharmacological drugs for AVS. However, future studies are encouraged to investigate changes in downstream Wnt/β-catenin gene expression in patients with AVS to corroborate our findings. Further investigations would be necessary to identify the stage in the Wnt pathway at which inhibition would be most effective in halting the progression of AVS.

## Data Availability Statement

The mass spectrometry proteomics data have been deposited to the ProteomeXchange Consortium via the PRIDE (61) partner repository with the dataset identifier PXD020783.

## Ethics Statement

The studies involving human participants were reviewed and approved by McGill University Health Centre. The patients/participants provided their written informed consent to participate in this study.

## Author Contributions

KK, BY, and AS designed the study. YS, SF, DS-T, and RC collected patient data and assisted with analysis. KK, BY, CK, and MC performed biological experimentation and analyzed the data. KK and AS wrote the manuscript. All authors have agreed upon the final draft of the manuscript.

## Conflict of Interest

The authors declare that the research was conducted in the absence of any commercial or financial relationships that could be construed as a potential conflict of interest.

## Supplementary Material

TABLE S1 Primers used in the RT-PCR analyses.

TABLE S2 Reference chart used for semi-quantitative analysis of immunohistochemistry experiments based on intensity and distribution.

TABLE S3 Reference chart used for aortic valve histpathology feature scoring.

TABLE S4 Semi-quantitative analysis of histological features.

TABLE S5 Semi-quantitative analysis of protein expression.
