# Supplementary material for: The Role of Wnt/β-Catenin Pathway Mediators in Aortic Valve Stenosis
Source: Front Cell Dev Biol. 2020 Sep 10;8:862. doi: 10.3389/fcell.2020.00862 (PMC7513845; doi:10.3389/fcell.2020.00862)
Supplement: TABLE S1 — Primers used in the RT-PCR analyses. [file Table_1.DOCX]

**Supplementary Table 1. Primers used in the RT-PCR analyses.**

| **Gene** | **Forward sequence** | **Reverse sequence** |
| --- | --- | --- |
| **BGLAP** | CATGAGAGCCCTCACACTCC | CTCCTGCTTGGACACAAAGG |
| **CTNNB1** | AAGGTGCTATCTGTCTGCTCTAGTA | TTCTGAACAAGACGTTGACTTGGAT |
| **DVL1** | TGGCATTGTCATCAAAGATCTCCTC | GTGCACGCCTACAAATTCTTCTTTA |
| **DVL2** | GGGCGTTGTCATCTGAAATTTCTTC | GGCGCCAAGTACTTTTTCAAGTCTA |
| **DVL3** | ATTGTCATCCGAGATCTCCTCCTTC | GCGACCCAGCTATAAGTTCTTCTTC |
| **GAPDH** | TGCACCACCAACTGCTTAGC | GGCATGGACTGTGGTCATGAG |
| **GSK3β** | CCACTGGAGTAGAAGAAATAACGCA | AACTGGTCGCCATCAAGAAAGTATT |
| **RUNX2** | ACCACAGAACCACAAGTGCG | CTGGTAGTGACCTGCGGAGA |
| **SFRP2** | CAACGACATAATGGAAACGCTTTGT | TCAGCTTGTAAATGGTCTTGCTCTT |
| **OPN** | ATTCTGGAAGTTCTGAGGAAAAGCA | CTAGGAGATTCTGCTTCTGAGATGG |
| **OSX** | AGAGAGGAGAGACTCGGGACAG | GACTGGAGCCATAGTGAACTTCCTC |
| **WNT3A** | ACTCGGATACTTCTTACTCCTCTGC | ACTCGGATACTTCTTACTCCTCTGC |
| **WNT5A** | CTTCCAAGTTCTTCCTAGTGGCTTT | TGAACAGGGTTATTCATACCTAGCG |
| **WNT5B** | GGAAACTGTCAGTCCCAGGGC | CTGCACCGGGTTCAAAGCTAAT |
| **WNT11** | AAGTTTTCCGATGCTCCTATGAAGG | ACTTACACTTCATTTCCAGAGAGGC |
