# Supplementary material for: The Role of Wnt/β-Catenin Pathway Mediators in Aortic Valve Stenosis
Source: Front Cell Dev Biol. 2020 Sep 10;8:862. doi: 10.3389/fcell.2020.00862 (PMC7513845; doi:10.3389/fcell.2020.00862)
Supplement: TABLE S2 — Reference chart used for semi-quantitative analysis of immunohistochemistry experiments based on intensity and distribution. [file Table_2.DOCX]

**Supplementary Table 2. Reference chart used for semi-quantitative analysis of immunohistochemistry experiments based on intensity and distribution.**

| **Score** | **Distribution** | **Intensity** |
| --- | --- | --- |
| **0** | No stain | No stain |
| **1** | <5% of ECM or cells | Faint |
| **2** | <25% of ECM or cells | Light brown |
| **3** | <50 of ECM or cells | Medium brown |
| **4** | <75% of ECM or cells | Deep brown |
| **5** | >75% of ECM or cells | Dark brown |
