# Supplementary material for: The Role of Wnt/β-Catenin Pathway Mediators in Aortic Valve Stenosis
Source: Front Cell Dev Biol. 2020 Sep 10;8:862. doi: 10.3389/fcell.2020.00862 (PMC7513845; doi:10.3389/fcell.2020.00862)
Supplement: TABLE S3 — Reference chart used for aortic valve histpathology feature scoring. [file Table_3.DOCX]

**Supplementary Table 3. Reference chart used for aortic valve histopathology feature scoring.**

| **Feature** | **Score: 0** | **Score: 1** | **Score: 2** | **Score: 3** | **Score: 4** |
| --- | --- | --- | --- | --- | --- |
| **Calcification** | None | Calcium deposition in < 25% of tissue | Calcium deposition in 25-50% of tissue | Calcium deposition in > 50% of tissue | N/A |
| **Fibrosis** | None | Section surface < 25% fibrosis | Section surface 25-50% fibrosis | Section surface >50% fibrosis | N/A |
| **Remodeling** | None | Mild fibrous thickening. Cusp structural integrity maintained | Moderate valve thickening. Some calcification. | Extensive thickening with a lot of calcification. | Extensive thickening and calcification. Destruction of most structural components |
| **Inflammation** | None | Occasional cells or 1 group with > 50 cells | 2-5 groups of > 50 cells | > 5 groups with > 50 cells or 1 group with > 500 cells | N/A |
