# Supplementary material for: The Role of Wnt/β-Catenin Pathway Mediators in Aortic Valve Stenosis
Source: Front Cell Dev Biol. 2020 Sep 10;8:862. doi: 10.3389/fcell.2020.00862 (PMC7513845; doi:10.3389/fcell.2020.00862)
Supplement: TABLE S4 — Semi-quantitative analysis of histological features. [file Table_4.DOCX]

**Supplementary Table 4. Semi-quantitative analysis of histological features.**

|  | **Control**  **Score** | **AVS**  **Score** | **P-value** |
| --- | --- | --- | --- |
| **Calcification** | 0.11 ± 0.07 | 2.67 ± 0.10 | < 0.0001 |
| **Fibrosis** | 1.39 ± 0.26 | 2.57 ± 0.10 | < 0.0001 |
| **Remodelling** (20) | 1.42 ± 0.17 | 3.37 ± 0.15 | < 0.0001 |
| **Inflammation** | 0.06 ± 0.06 | 0.87 ± 0.16 | 0.0038 |
