# Supplementary material for: The Role of Wnt/β-Catenin Pathway Mediators in Aortic Valve Stenosis
Source: Front Cell Dev Biol. 2020 Sep 10;8:862. doi: 10.3389/fcell.2020.00862 (PMC7513845; doi:10.3389/fcell.2020.00862)
Supplement: TABLE S5 — Semi-quantitative analysis of protein expression. [file Table_5.DOCX]

**Supplementary Table 5. Semi-quantitative analysis of protein expression**

|  | **Control**  **Score** | **AVS**  **Score** | **P-value** |
| --- | --- | --- | --- |
| **DVL2** | 0.39 ± 0.12 | 2.57 ± 0.16 | < 0.0001 |
| **GSK-3**$\boldsymbol{\beta}$ | 0.33 ± 0.11 | 2.94 ± 0.14 | < 0.0001 |
| **CTNNB1** | 0.56 ± 0.18 | 2.33 ± 0.29 | 0.0006 |
| **SFRP2** | 0.44 ± 0.15 | 3.00 ± 0.14 | < 0.0001 |
